# Potential Association between Kaposi Sarcoma and Gout: An Exploratory Observational Study

**DOI:** 10.1155/2020/8844970

**Published:** 2020-12-01

**Authors:** Assaf Moore, Idit Peretz, Lilach Yosef, Daniel A. Goldstein, Hadar Goldvaser, Suzanna Horn, Yonatan Edel, Alona Zer

**Affiliations:** ^1^Institute of Oncology, Davidoff Center, Rabin Medical Center—Beilinson Hospital, Petach Tikva, Israel; ^2^Sackler Faculty of Medicine, Tel Aviv University, Tel Aviv, Israel; ^3^Pathology Department, Rabin Medical Center—Beilinson Hospital, Petach Tikva, Israel; ^4^Rheumatology Unit, Rabin Medical Center—Beilinson Hospital, Petach Tikva, Israel; ^5^Medicine C, Rabin Medical Center—Beilinson Hospital, Petach Tikva, Israel

## Abstract

**Background:**

Kaposi sarcoma is a rare vascular mesenchymal neoplasm, associated with Human Herpes Virus 8 (HHV8). Gout is a condition clinically characterized by recurrent flares of arthritis and hyperuricemia. Following our clinical impression that patients with classical Kaposi sarcoma (CKS) have a high rate of gout, we explored this in a retrospective manner.

**Methods:**

All consecutive patients diagnosed with sarcoma or carcinosarcoma within a single tertiary center between 1/2012–12/2017 were identified through the pathology department database. A cohort of CKS patients was compared with the non-Kaposi sarcoma and carcinosarcoma cohort. Data were extracted from patients' electronic medical records. Patients younger than 18 and patients without clinical data available were excluded. Association between diagnosis of gout and CKS was assessed and adjusted for risk factors.

**Results:**

Three hundred and sixty-one patients were eligible for this analysis, 61 were diagnosed with CKS and 300 with other types of sarcoma. We found a higher incidence of gout in CKS patients, 11/61 (18%) patients, compared with 8/300 (2.6%) with other types of sarcoma, odds ratio (OR) 8.0 (*P* < 0.00001). This association persisted when adjusted for age >39 years (OR = 6.7, *P* < 0.00001), age and male sex (OR = 4.97, *P* < 0.0001), and when adjusting for multiple confounding factors and medical comorbidities.

**Conclusions:**

We have demonstrated a statistically significant association between gout and CKS. As risk factors for gout were accounted for, this association may be explained by HHV8 immune-related effects. This should be further explored in vitro and in population-based studies.

## 1. Background

Kaposi sarcoma (KS) is an angioproliferative disorder, originating from endothelial cells, myofibroblasts, and monocyte-macrophage cells. Four subtypes have been identified: classical KS (CKS); endemic, described in sub-Saharan indigenous Africa; iatrogenic, associated with immunosuppression; and AIDS associated. While KS has been described globally, the disease is most frequent in Mediterranean populations and Central and Eastern Europe [1–4].

KS was found to have distinct risk and etiological factors. KS is more frequent in men and usually presents in the seventh and eight decades of life. Only 4–8% of patients present under the age of 50 years [5, 6]. KS was found to be strongly associated with human herpes virus 8 (HHV-8) infection [7]. Its incidence was found to be 20,000 times more frequent in patients with AIDS compared with the general population and 300 times more frequent compared with other immune-suppressed patient groups [8]. It is more commonly associated with HIV-1 infection than with HIV-2 infection [9]. KS was also found to be associated with anemia [10], topical and oral corticosteroid use [11, 12], infrequent bathing [12], lower extremity edema, and diabetes mellitus [11]. A lower risk of KS was found in cigarette smokers [12].

Gout is characterized by hyperuricemia and monosodium urate (MSU) crystal deposition, causing flares of acute inflammatory arthritis, chronic arthropathy, tophaceous deposits of urate crystals, and nephrolithiasis. The incidence increases in men in the fourth and fifth decades of life and in the sixth or seventh decades in women [13, 14]. Known risk factors for gout are renal function impairment, decreased excretion through the gastrointestinal system and urate overproduction, obesity, and alcohol consumption [15–17]. The prevalence of gout in the overall US adult population approaches 4% and is in the region of 6% in men and 2% in women [18]. One study found that the prevalence of gout in the US varies by body mass index (BMI) and ranged between 1-2% in patients with a normal BMI and up to 5–7% in morbidly obese [19].

A correlation between KS and gout has been explored before without definite conclusions [11, 12].

Following a clinical impression in our clinics that patients with CKS have a high rate of gout, we set out to explore this finding in a large cohort of patients with CKS compared with a control cohort of patients with other sarcomas/carcinosarcomas.

## 2. Methods

### 2.1. Patients

The registry of the pathology department at the Rabin Medical Center was screened for all patients diagnosed with sarcomas/carcinosarcomas between 1/2012–12/2017. Patients with a diagnosis of CKS comprised one cohort, and patients diagnosed with all other sarcomas or carcinosarcomas comprised the “control” cohort. Patients' demographics and clinicopathological characteristics were extracted from electronic hospital and community-based medical records. Patients younger than 18 years of age and patients without available clinical data were excluded from this analysis. The study was approved by the institutional ethics committees prior to any research procedures.

### 2.2. Statistics Analysis

Association between diagnosis of gout and CKS was calculated and adjusted for risk factors including age, sex, and medical comorbidities using a logistic regression test via MEDCALC® statistical software [20]. Associations were presented with odds ratio (OR) and 95% confidence interval (CIs). A significance level of 0.05 was used. The *χ*2 test was used for categorical data, and the Student's *t*-test was used for continuous data.

## 3. Results

A total of 700 patients with a pathological diagnosis of sarcoma or carcinosarcoma were located in the registries. After exclusion of 339 cases not meeting the inclusion criteria, 361 patients were eligible for this analysis and comprised the study cohort. Of them, 61 patients were diagnosed with CKS and comprised the study group, and 300 patients with other types of sarcoma or carcinosarcoma comprised the control group.

The CKS cohort was significantly older than the general sarcoma cohort and had a larger proportion of male patients, as well as a significantly higher proportion of medical comorbidities, including hypertension, diabetes mellitus, and solid organ transplantation. There was no significant difference in oral steroid use. We found a higher incidence of gout in CKS patients, 11/61 (18%), compared with 8/300 (2.6%) with other types of sarcoma and carcinosarcoma (OR = 8.0, 95% CI 3.08–20.94, *P* < 0.00001), as well as a more common use of allopurinol in patients with CKS. Patient characteristics are available in Table 1.

The association between CKS and gout remained significant when adjusting for single confounding factors, such as age at diagnosis >39 years (OR 6.7, 95% CI 2.4–18.1, *P* < 0.0001), male sex (OR 6, 3, 95% CI 2.4–16.7, *P* < 0.0001), diabetes mellitus (OR 9.1, 95% CI 3.4–24.3, *P* < 0.0001), and oral steroid use (OR 8.2, 95% CI 3.1–21.5, *P*=0.0001).

When adjusting for both age (>39 years) and male sex, the OR was 5.0 (95% CI 1.8–13.7, *P* < 0.0001). The association also remains significant when adjusting for multiple factors, such as age at diagnosis, sex, and hypertension (OR 5.0, 95% CI 1.8–14.0, *P* < 0.0001) and age at diagnosis, sex, hypertension, diabetes mellitus, and oral steroid use (OR 7.2, 95% CI 2.6–20.1, *P* < 0.0001).

## 4. Discussion

This study identified a statistically significant association between gout and CKS, as compared with a cohort of patients with non-Kaposi sarcomas and carcinosarcomas. As expected, the use of allopurinol was more prevalent among patients with CKS. While the CKS cohort had more risk factors for gout, including older age, a higher prevalence of male patients and medical comorbidities was known to be associated with gout, when adjusting for these confounders separately and in conjunction, and the significant association remained.

Two studies have previously reported on such a possible association. In one study, a higher incidence of gout was found in a cohort of classical KS compared with HHV8-positive controls, 14/142 vs. 8/123 patients, OR 1.28 (0.45–3.62) [11]. The CKS cohort had a higher prevalence of diabetes mellitus, kidney failure, and oral steroid use compared with a randomly selected sex and age-matched HHV8-seropositive cohort [11]. That study did not report on organ transplantation. In the second study, a CKS cohort was also compared with an HHV8-seropositive control cohort of the same age and sex and from the same communities. That study also reported an increased risk for KS with a history of gout (OR = 2.13, 95% CI = 0.74 to 6.15), a higher prevalence of steroid use in CKS patients, no excess kidney failure, and no cases of organ transplantation [12]. In both studies, the higher prevalence of gout in CKS patients was not statistically significant [11, 12]. The median age in these studies was similar to that of our CKS cohort; however, we found no difference in the prevalence of steroid use, and we did not account for kidney function. We selected a non-Kaposi cancer-control cohort in this study to overcome a potential causation with malignancy or paraneoplastic phenomenon. Our results are strengthened by the fact that our non-Kaposi cancer-control cohort had a similar prevalence of gout compared with rates reported in the literature [18].

While our results are not in accordance with these previous reports, potential explanations for this association can be considered. HHV8 can establish a lifelong infection. In order to enter the latent phase, the virus modulates the host's innate immune response [21–23]. Toll-like receptors (TLRs) are transmembrane proteins that are key components of the innate immune system's ability to recognize invading pathogens. TLRs 2, −3, −4, −7, −8, and −9 have been recognized to be involved in the recognition of viruses [24–26]. HHV8 is known to have complex interactions with TLRs resulting in their activation. One study found that, through infection of THP-1- and CD14-positive primary human monocytes, HHV8 upregulates the TLR3 pathway and induces TLR3-specific cytokines and chemokines, including beta 1 interferon (IFN-*β*1) and CXCL10 (IP-10) [27]. CXCL10 is an important chemokine that can regulate immune responses through the activation and recruitment of immune cells [28, 29]. CXCL10 has been shown to be involved in the development of arthritis [30].

A gout flare is characterized by an acute inflammatory response that occurs in the synovium by urate crystal deposition or release. Neutrophil phagocytosis of crystals in the aspirated synovial fluid is the hallmark for diagnosis of gout [31]. However, many other inflammatory processes occur prior to and during neutrophil activation. Gout-related inflammation is thought to be related in part to TLR-dependent inflammatory response. One study showed that, in patients with gout, hyperuricemia causes a shift in the IL-1*β*/IL-1Ra balance produced by peripheral blood mononuclear cells (PBMCs) after exposure to MSU crystals and TLR (3 and 4 ligand)-mediated stimuli, and this phenomenon is likely to reinforce the enhanced state of chronic inflammation [32]. In another study, monocytes from healthy volunteers were primed with uric acid, leading to increased activity in mammalian target of rapamycin (mTOR) signaling. This led to lower autophagic activity in cells exposed to uric acid compared with control conditions and diminished reactive oxygen species production [33].

TLRs and CXCL10 may provide a partial explanation to the potential association between CKS and gout, as the first was shown to upregulate an important TLR in gout, as well as an important chemokine to the development of arthritis. In fact, drugs targeting CXCL10 may be a new therapeutic approach for acute gouty arthritis [30, 34].

Furthermore, a study demonstrated how HHV8 infection involved with KS interacts with procaspase-1 to form functional inflammasomes [35]. The inflammasome acts as a sensor for various types of molecules in the cytoplasm, including RNA viruses, and is responsible for the activation of inflammatory responses of the innate immune system [36]. In gout, the IL-1-dependent innate inflammatory phenotype is now known to rely on the formation of the macromolecular NLRP3 inflammasome complex in response to the MSU [37]. This common feature of inflammasome activation may also contribute to a common pathophysiology.

The main limitations of this study relate to its retrospective design that is generally associated with methodological biases, especially selection bias, and difficulties in interpreting results. The study strengths include consecutive patient enrolment and being a relatively large size single institution cohort. Another strength is the availability of community-based patient follow-up on joint databases, which allows for better patient follow-up and documentation of comorbidities.

## 5. Conclusions

We have demonstrated a statistically significant association between CKS and gout. As risk factors for gout were accounted for, this association may be explained by HHV8 immune-related effects. This should be further explored in vitro and in population-based studies.

## Figures and Tables

**Table 1:** Patients' characteristics, *n* = 366.

Factor	CKS cohort, *n* = 61	General sarcoma cohort, *n* = 300	*P* value	OR
Median age, years (range)	78 (39–94)	68 years (39–99)	<0.0001 (95% CI 4.97–12.20)	—
Male sex, *n* (%)	46 (75.4%)	155 (51.7%)	<0.0007	2.9, 95% CI 1.53–5.36
Hypertension, *n* (%)	42 (68.8%)	168 (56%)	0.06	1.7, 95% CI 0.96–3.13
Diabetes mellitus, *n* (%)	24 (39.3%)	88 (29.3%)	0.12	1.6, 95% CI 0.88–2.76
Oral steroid use, *n* (%)	13 (21.3%)	70 (23.3%)	0.73	0.89, 95% CI 0.45–1.74
Gout, *n* (%)	11 (18%)	8 (2.6%)	<0.00001	8.0, 95% CI 3.08–20.94
Regular allopurinol use, *n* (%)	10 (16.4%)	10 (3.3%)	<0.0001	5.7, 95% CI 2.25–14.34

CKS, classical Kaposi sarcoma; *n*, number; OR, odds ratio.

## Data Availability

The data used to support the findings of this study are available from the corresponding author upon request.
